# Vpr Enhances HIV-1 Env Processing and Virion Infectivity in Macrophages by Modulating TET2-Dependent IFITM3 Expression

**DOI:** 10.1128/mBio.01344-19

**Published:** 2019-08-20

**Authors:** Qi Wang, Lishan Su

**Affiliations:** a Lineberger Comprehensive Cancer Center, University of North Carolina at Chapel Hill, Chapel Hill, North Carolina, USA; b Department of Microbiology & Immunology, The University of North Carolina at Chapel Hill, Chapel Hill, North Carolina, USA; Vanderbilt University Medical Center; University of Pittsburgh School of Medicine

**Keywords:** demethylation, Env processing, HIV-1 infectivity, IFITM3, TET2, Vpr

## Abstract

HIV-1 Vpr enhances viral replication in human macrophages via multiple mechanisms that are not clearly defined. It does not affect HIV-1 virion production during the first round of infection. We have recently discovered that Vpr targets the DNA demethylase TET2 for degradation, which leads to sustained interleukin-6 (IL-6) expression and elevated HIV-1 replication. We report here that Vpr enhanced Env processing in infected macrophages, associated with increased Env incorporation into virions with higher infectivity. Interestingly, IFITM3 was constitutively expressed in macrophages in a TET2-dependent fashion. We showed that Vpr-enhanced Env processing depended genetically on TET2 and IFITM3. We further showed that Vpr reduced IFITM3 expression by reducing demethylation of the IFITM3 promoter in macrophages, associated with degradation of TET2 and reduced TET2 binding to the IFITIM3 promoter. Our findings indicate that the Vpr-TET2 axis enhances HIV-1 replication in macrophages via two independent mechanisms: reduced IFTIM3 expression to enhance Env processing and virion infectivity and sustained IL-6 expression to increase HIV-1 replication. The Vpr-TET2 axis may provide a novel target to develop therapeutics to inhibit HIV-1 infection and pathogenesis.

## Abstract (precis)

How Vpr enhances HIV-1 replication in macrophages is still unclear. We report here that Vpr enhanced HIV-1 Env processing during the first round of HIV-1 replication, resulting in virions with higher Env incorporation and viral infectivity. These higher-quality viral particles contributed to elevated infection during the second round and spreading infection in macrophages and other HIV-1 target cells. We have recently discovered that TET2 is a novel host factor degraded by Vpr, which leads to sustained IL-6 expression in macrophages. Interestingly, Vpr-enhanced HIV-1 Env processing depended on both the IFITIM3 and TET2 genes. The constitutive expression of IFITIM3 expression in macrophages was maintained by TET2, which demethylated the IFITIM3 promoter. We conclude that the Vpr degrades TET2 to enhance HIV-1 replication in macrophages by reducing IFITIM3 expression to increase viral Env processing, virion incorporation, and infectivity and by sustaining IL-6 expression to increase HIV-1 gene expression. The Vpr-TET2 axis may serve as a novel target to develop anti-HIV drugs to inhibit HIV-1 infection and pathogenesis.

## INTRODUCTION

HIV-1 infection of macrophages contributes to its persistent infection in HIV-1 patients with highly active antiretroviral therapy (HAART), bringing complexity to viral eradication for HIV-1 cure (1). Although macrophages are relatively resistant to HIV-1 infection compared to activated CD4^+^ T cells (2), HIV-1 has evolved to efficiently infect macrophages, which is enhanced by HIV-1 viral proteins such as viral protein R (Vpr) to counteract macrophage-specific restrictions (3, 4). Vpr, a small 14-kDa protein, is functionally critical for HIV-1 pathogenicity *in vivo* (5). Vpr has been reported to promote infection of nondividing cells, especially macrophages (6). However, when virus entry, reverse transcription (RT), integration, gene expression, virion assembly and budding, or total virions are measured, Vpr does not affect the first-round replication of HIV-1 in macrophages. Multiple mechanisms seem to contribute to Vpr-enhanced HIV-1 replication, including more infectious virions and cytokine-dependent effects (7, 8).

The exact mechanism of Vpr-enhanced HIV-1 replication in macrophages, however, remains elusive. Vpr is believed to facilitate HIV-1 replication through its interaction with VprBP (9), a most abundant CUL4 binding partner first discovered by its binding with Vpr (also known as DCAF1) (10, 11). Several host proteins have been reported to be targeted by Vpr for ubiquitylation by the CRL4^VprBP^ E3 ligase, including MCM10 (12), UNG2 (13), and MUS81 (14). Recently, we discovered that Vpr targets the DNA demethylase TET2, which functions as a repressor to resolve induction of the interleukin-6 (IL-6) gene in HIV-1-infected macrophages (15). TET2 deactivates gene expression through recruitment of the histone deacetylase (HDAC) complex to promoter DNA (16). In macrophages, Vpr-induced TET2 depletion prevents efficient resolution of IL-6 induction during HIV-1 infection, which enhances HIV-1 infection in macrophages. Interestingly, the TET2 dioxygenase activity is not required for the suppression of IL-6 gene expression during its resolution phase (16).

In mammalian cells, the majority of CpG dinucleotides outside the CpG islands (CGIs) are methylated at the C-5 position of cytosine (5mC) throughout the genome to stably maintain intergenic and heterochromatic regions in a transcriptionally inert chromatin state. CGIs, on the other hand, are associated with many (∼70%) promoters (17) and, when methylated, are associated with gene silencing. TET methylcytosine dioxygenases (TET1, -2, and -3 in mammalian cells [18, 19]) catalyze three steps of iterative oxidation, first converting 5mC to 5-hydroxymethyl cytosine (5hmC), then 5hmC to 5-formyl cytosine (5fC), and finally 5fC to 5-carboxy cytosine (5caC). 5caC can be removed by DNA glycosylase TDG, resulting in 5-unmodified cytosine (20, 21). TET2 is thus a dioxygenase that catalyzes oxidative decarboxylation of α-KG, creating a highly reactive intermediate that converts 5mC to 5hmC (22) and activates gene expression through promotion of DNA demethylation of their promoters (23).

We report here that Vpr enhanced Env processing, associated with increased HIV-1 infectivity during the first round of infection in macrophages. Vpr-enhanced Env processing depended genetically on TET2 and IFITM3, which is constitutively expressed in macrophages in a TET2-dependent fashion. We further showed that Vpr reduced IFITM3 expression by degrading TET2 in macrophages, associated with reduced demethylation of the IFITM3 promoter. We demonstrate that the Vpr-TET2 axis enhanced HIV-1 replication in macrophages via two independent mechanisms: (i) reduced IFTIM3 expression to enhance Env processing and virion infectivity and (ii) sustained IL-6 expression to increase HIV-1 replication.

## RESULTS

### Vpr enhances HIV-1 Env processing and virion infectivity during the first round of replication in macrophages.

**FIG 1:**
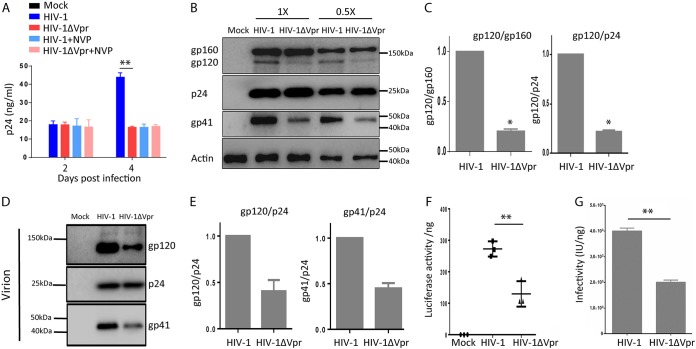
Vpr enhances Env processing and virion infectivity in MDMs. (A) Vpr has no effect on first-round HIV-1 replication in macrophages. MDMs were infected with HIV-1 or HIV-1 ΔVpr viruses (MOI = 0.1). Levels of p24 in the supernatant were assessed at 2 and 4 dpi. Cells were treated with 2 μM nevirapine (NVP) at 2 dpi, where indicated, to inhibit the second round of HIV-1 infection. (B) Vpr enhances HIV-1 Env processing in macrophages. Shown are Western blots of cell lysates from MDMs infected with HIV-1 for 4 days, with NVP added at 2 dpi. Env proteins gp160 and gp120 were detected with anti-gp120 antibody, gp41 was detected by anti-gp41 antibody, p24 was detected by anti-p24 antibody, and actin was detected by antiactin antibody. Samples diluted 1× or 2× were tested as indicated. (C) Vpr-enhanced gp120/gp160 and gp120/p24 ratios in MDMs are summarized with MDMs from three independent experiments with three different donors. (D) Vpr enhances Env incorporation in virions from MDMs. Viral supernatants were concentrated from MDMs infected with HIV-1 or HIV-1 ΔVpr viruses at 2 dpi by ultracentrifugation (over 20% sucrose). Western blotting was performed by anti-gp120, -p24, and -gp41 antibodies. (E) Vpr-enhanced gp120/p24 ratios and gp41/p24 ratios in virions produced from MDMs are summarized from two different donors. (F and G) Vpr enhances specific infectivity of virions from MDMs. (F) Supernatants with equal amounts of p24 from MDMs infected with wild-type HIV-1 or HIV-1 ΔVpr viruses were used to infect TZM-bl indicator cells. Luciferase activity was measured at 48 hpi. Data (luciferase/ng p24) from three wells were used from each donor MDM, and 3 technical repeats were performed with supernatant from each well. MDMs derived from 3 donors (*n* = 3) were used to repeat the experiments. Shown are the mean values of MDMs from 3 donors. **, *P* < 0.01 by nonparametric one-way analysis of variance (ANOVA). (G) Supernatants with equal amounts of p24 from MDMs infected with wild-type HIV-1 or HIV-1 ΔVpr viruses were used to infect indicator MAGI-CCR5 cells. Infectivity represents infectious units (IU) per ng p24. Values are average IU/p24 from three independent experiments with MDMs from three donors.

We investigated the role of Vpr in enhancing HIV-1 replication in human primary macrophages. As previously reported (6), we observed that macrophage-tropic Vpr^+^ HIV-1 or Vpr^−^ HIV-1 infected and replicated to similar levels during the first cycle of infection at 2 days postinfection (dpi) in monocyte-derived macrophages (MDMs). However, Vpr^+^ HIV-1 showed elevated levels of replication at 4 dpi as determined by HIV-p24 enzyme-linked immunosorbent assay (ELISA) (Fig. 1A) or by intracellular HIV-1 p24 staining (see Fig. S1 in the supplemental material). To confirm that the first cycle of HIV-1 replication was not affected by Vpr, we added reverse transcriptase inhibitor nevirapine (NVP) at 2 dpi to block second-round HIV-1 infection. We found that Vpr enhanced viral replication at 4 dpi, but failed to do so when NVP was added at 2 dpi (Fig. 1A and Fig. S1).

10.1128/mBio.01344-19.1 FIG S1 MDMs were infected with HIV-1 or HIV-1 ΔVpr viruses at an MOI of 0.1. Cells were treated with 2 μM nevirapine (NVP) at 2 dpi where indicated. (A) Fluorescence-activated cell sorter (FACS) analysis of the percentage of infected (p24^+^) cells at 4 dpi. (B) The percentages of p24^+^ cells are summarized (*n* = 2 donors). For related details, see Fig. 1. Download FIG S1, PDF file, 0.5 MB. Copyright © 2019 Wang and Su. 2019 Wang and Su This content is distributed under the terms of the Creative Commons Attribution 4.0 International license.

To explore the mechanism by which Vpr enhances second-round viral replication, we examined processing of HIV-1 viral proteins in MDMs infected with Vpr^+^ or Vpr^−^ HIV-1 during first-round infection. As shown in Fig. 1B and C, the ratio of gp120 to gp160 or gp120/p24 in Vpr^+^ HIV-1 infected MDMs was ∼ 5-fold higher than that of MDMs infected with Vpr^−^ HIV-1. We confirmed the results with MDMs derived from three different donors, and the enhanced ratio of gp120 to gp160 or gp120/p24 by Vpr is summarized in Fig. 1C. Consistently, HIV-1 virions produced from MDM infected with Vpr^+^ HIV-1 showed 3-fold-higher levels of HIV-1 Env proteins (Fig. 1D and E), as previously reported (7). When the relative infectivity was measured, HIV-1 virions produced from MDM infected with Vpr^+^ HIV-1 showed 2.5-fold-higher infectivity by TZM-bl luciferase indicator assay (Fig. 1F, relative luciferase activity/ng p24), or by the MAGI (multinuclear activation of galactosidase indicator) infectious unit (IU) assay. We showed that Vpr^+^ HIV-1 virions contained 4 × 10^3^ IU/ng p24 and Vpr^−^ HIV-1 virions contained 2 × 10^3^ IU/ng p24 (Fig. 1G, relative IU/ng p24). We further showed that virions produced from MDMs infected with Vpr^+^ HIV-1 were more resistant to inhibition by soluble CD4 (sCD4) than Vpr^−^ HIV-1 virions produced from the first round of viral replication in MDMs. Vpr^−^ HIV-1 virions were ∼10× more sensitive to sCD4 inhibition, with a 50% inhibitory concentration (IC_50_) of 0.034 μg/ml, whereas Vpr^+^ HIV-1 virions were relatively refractory to sCD4 inhibition, with an IC_50_ of 0.366 μg/ml (see Fig. S2 in the supplemental material). These results indicate that Vpr enhances Env processing during HIV-1 infection in MDM to produce more infectious HIV-1 virions with higher Env incorporation from MDMs.

10.1128/mBio.01344-19.2 FIG S2 TZM-bl indicator cells were infected with equal amounts of p24 of HIV-1 or HIV-1 ΔVpr viruses incubated with different doses of sCD4. Levels of HIV-induced luciferase for each virus stock without sCD4 treatment are defined as 100%. The IC_50_ was calculated (μg/ml). (A) HIV-1 virus. (B) HIV-1 ΔVpr virus. For related details, see Fig. 1. Download FIG S2, PDF file, 0.03 MB. Copyright © 2019 Wang and Su. 2019 Wang and Su This content is distributed under the terms of the Creative Commons Attribution 4.0 International license.

### IFITM3 expression in MDMs is dependent on TET2.

**FIG 2:**
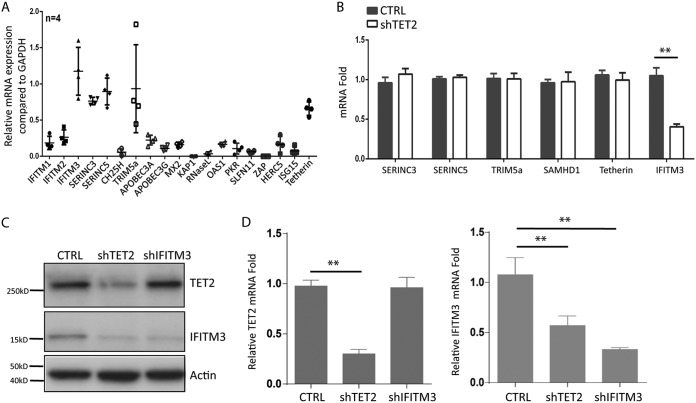
IFITM3 is constitutively expressed in MDMs via TET2-dependent mechanisms. (A) Relative gene expression of HIV-1 restriction factors in MDMs derived from 4 donors is summarized. (B) MDMs were transduced with lentiviral vectors encoding control shRNA or with TET2 shRNA. Relative expression of restriction factors with detectable expression in MDMs, including Serinc3, Serinc 5, Trim 5a, SAMHD1, tetherin, and IFITM3, was measured. (C) TET2 and IFITM3 were detected with anti-TET2 antibody and anti-IFITM3 antibody by Western blotting with cell lysates from MDMs transduced with control shRNA, TET2 shRNA, and IFITM3 shRNA. (D) TET2 (left panel) and IFITM3 (right panel) mRNAs were detected by RT-qPCR in MDMs transduced with control, TET2, and IFITM3 shRNA. * and ** indicate *P* < 0.05 and *P* < 0.01, respectively.

To investigate host factors that may inhibit HIV-1 replication in human MDMs, we measured relative gene expression of known anti-HIV host factors. Interestingly, 5 of the 19 human genes were constitutively expressed in resting MDMs (Fig. 2A). We recently reported that Vpr degrades TET2 through the CRL4^Vprbp^ E3 ligase in macrophages (15). We analyzed the relative expression of known HIV-1 restriction factors in MDMs transduced with control or TET2 short hairpin RNA (shRNA). Although 6 genes encoding anti-HIV host factors were expressed in MDMs (Fig. 2A and B), only IFITIM3 was expressed in a TET2-dependent fashion at both mRNA and protein levels (Fig. 2B to D).

### Vpr enhances Env processing/virion infectivity via TET2- and IFITM3-dependent mechanisms.

**FIG 3:**
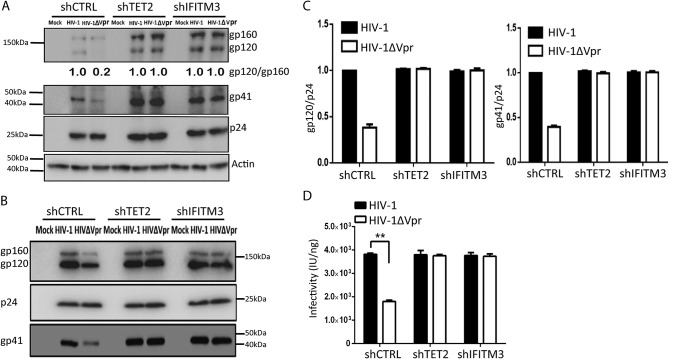
Vpr enhances Env processing/infectivity in MDMs via TET2- and IFITM3-dependent mechanisms. (A) MDMs transduced with lentiviral vectors with shRNA-mediated knockdown of TET2 and IFITM3 were infected with HIV-1 or HIV-1 ΔVpr viruses as described above. Western blot analysis of cell lysates was performed as in Fig. 1B. The gp120/gp160 ratio of HIV-1 from control MDMs is defined as 100%. (B) MDMs were transduced with lentivirus vectors with control shRNA, shTET2, and shIFITM3. Viral particles were purified by ultracentrifugation (over 20% sucrose) from the aforementioned MDMs infected with HIV-1 or HIV-1 ΔVpr viruses for 4 days, with NVP added at 2 dpi. Five nanograms of p24 per virion for each sample was added per lane for analysis of gp120, p24, and gp41 in virions. Western blotting was performed by using anti-gp120, p24, and gp41 antibodies. (C) The ratios of gp120/p24 and of gp41/p24 are summarized from three different donors. (D) Supernatants from MDMs infected with HIV-1 or HIV-1 ΔVpr viruses were used to infect TZM-bl indicator cells. The relative infectivity is defined as HIV-induced luciferase/ng (*n* = 3 donors).

IFITM3 has been reported to inhibit virion assembly of various viruses, including HIV-1 (24, 25). IFITM3 has been recently reported to inhibit Env processing in HIV-1-infected target cells (24). This suggests that the Vpr-TET2 axis may modulate IFITM3 expression to enhance HIV-1 Env processing and virion infectivity in MDMs. We thus depleted TET2 or IFITM3 through shRNA-mediated knockdown in MDMs to explore whether Vpr enhanced Env processing through these two proteins (see Fig. S3A in the supplemental material). The ratio of gp120 to gp160 in control MDMs infected with Vpr^+^ HIV-1 was 5-fold higher than that of Vpr^−^ HIV-1. In MDMs with depletion of TET2 or IFITM3, both Vpr^+^ HIV-1 and Vpr^−^ HIV-1 showed a similar level of Env processing (Fig. 3A). Vpr failed to further enhance the ratio of gp120 to gp160 in HIV-1-infected MDMs with depletion of TET2 or IFITM3 (Fig. 3A). IFITM3 in target cells has been reported to inhibit HIV-1 entry (26). Consistently, predepletion of TET2 or IFITM3 led to elevated entry of both Vpr^+^ HIV-1 and Vpr^−^ HIV-1 (Fig. 3A; Fig. S3B and C). We further measured Env levels in HIV-1 virions produced from MDMs with depletion of TET2 and IFITM3. HIV-1 virions produced from control MDMs infected with Vpr^+^ HIV-1 showed 3-fold-higher levels of Env proteins than Vpr^−^ HIV-1 virions. Consistent with the elevated Env processing, Vpr^+^ HIV-1 and Vpr^−^ HIV-1 virions from MDMs with depletion of TET2 or IFITM3 contained equal amounts of Env proteins (Fig. 3B and C). Moreover, Vpr^+^ or Vpr^−^ HIV-1 virions produced from MDMs with depletion of TET2 or IFITM3 showed equal infectivity measured by TZM-bl luciferase assay and by MAGI IU assay (Fig. 3E; Fig. S3D). Our data suggest that the TET2-IFITM3 axis enhances HIV-1 Env processing and virion infectivity in MDMs.

10.1128/mBio.01344-19.3 FIG S3 MDMs with shRNA-mediated knockdown of TET2 and IFITM3 were infected with HIV-1 or HIV-1 ΔVpr viruses. Cells were treated with 2 μM nevirapine (NVP) at 2 dpi. (A) TET2 (left) and IFITM3 (right) were knocked down by shRNA in MDMs, and the knockdown efficiency was confirmed by RT-qPCR. (B) Levels of p24 in the supernatant were assessed at 4 dpi. (C) MDMs with shRNA-mediated knockdown of TET2 and IFITM3 were infected with HIV-1 or HIV-1 ΔVpr viruses. DNA was harvested at 3 h postinfection for viral entry analysis by qPCR for strong-stop HIV DNA (R-U5). (D) Supernatants from MDMs infected with HIV-1 or HIV-1 ΔVpr viruses were used to infect TZM-bl indicator cells. The relative infectivity was measured by HIV-induced luciferase activity per ng of p24. Supernatant from control MDMs infected with HIV-1 is defined as 100% (*n* = 3 donors). For related details, see Fig. 3. Download FIG S3, PDF file, 0.3 MB. Copyright © 2019 Wang and Su. 2019 Wang and Su This content is distributed under the terms of the Creative Commons Attribution 4.0 International license.

**FIG 4:**
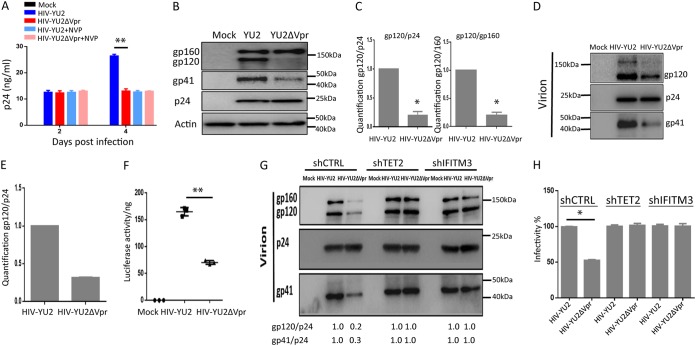
The Vpr-TET2 axis enhances HIV-Yu-2 Env processing and virion infectivity in MDMs. (A) MDMs were infected with Yu-2 or Yu-2 ΔVpr viruses (MOI = 0.1). Levels of p24 in the supernatant were assessed at 2 and 4 dpi. Cells were treated with 2 μM nevirapine (NVP) at 2 dpi where indicated, to inhibit the second round of HIV-Yu-2 infection. (B) Western blots of cell lysates from MDMs infected with Yu-2 or Yu-2 ΔVpr for 4 days with NVP added at 2 dpi. Env proteins gp160 and gp120 were detected with anti-gp120 antibody, gp41 was detected with anti-gp41 antibody, and p24 was detected with anti-p24 antibody. (C) Vpr-enhanced gp120/p24 and gp120/gp160 ratios in MDMs are summarized with MDMs from with two different donors. (D) Viral supernatants were concentrated from MDMs infected with Yu-2 or Yu-2 ΔVpr viruses at 2 dpi by ultracentrifugation (over 20% sucrose). Western blotting was performed by using anti-gp120, -p24, and -gp41 antibodies. (E) Vpr-enhanced gp120/p24 ratios are summarized from two different donors. (F) Supernatants with equal amounts of p24 from MDMs infected with Yu-2 or Yu-2 ΔVpr viruses were used to infect TZM-bl indicator cells. Luciferase activity was measured at 48 hpi. Data (luciferase/ng p24) from three wells were used from each donor MDM, and 3 technical repeats were performed with supernatant from each well. MDMs derived from 2 donors were used to repeat the experiments. (G) MDMs were transduced with control shRNA, shTET2, and shIFITM3. Viral supernatants were concentrated from the aforementioned MDMs infected with Yu-2 or Yu-2 ΔVpr viruses for 4 days, with NVP added at 2 dpi, by ultracentrifugation (over 20% sucrose). Five nanograms of p24 for each virion was added per lane for analysis of gp120, p24, and gp41 in virions. (H) Supernatants from MDMs infected with Yu-2 or Yu-2 ΔVpr viruses were used to infect TZM-bl indicator cells. The relative infectivity is defined by HIV-induced luciferase activity per ng of p24. Supernatant from control MDMs infected with HIV-YU-2 is defined as 100% (*n* = 2 donors).

To further confirm that Vpr enhances Env processing/virion infectivity via TET2- and IFITM3-dependent mechanisms in macrophages, we generated and tested a primary Vpr^+^ and Vpr^−^ macrophage/CCR5-tropic HIV-1 clone. MDMs were infected with HIV-Yu-2 and HIV-Yu-2 ΔVpr viruses. Vpr showed no effect on the first-round HIV-1 replication (Fig. 4A). Vpr similarly enhanced Yu-2 Env processing in MDMs (Fig. 4B and C), and Env incorporation in virions with elevated infectivity (Fig. 4D to F). As expected, the Vpr-mediated enhancement of Env processing in MDM cells, Env levels in virions, and virion infectivity in MDMs depended on TET2 and IFITM3 (Fig. 4G and H). Therefore, Vpr enhances Env processing/virion infectivity in a TET2- and IFITM3-dependent manner in macrophages with both CXCR4/CCR5 dualtropic (HIV-R3A) and CCR5-tropic (Yu-2) HIV-1 strains.

### Vpr degrades TET2 to prevent demethylation of the IFITM3 promoter in MDMs.

**FIG 5:**
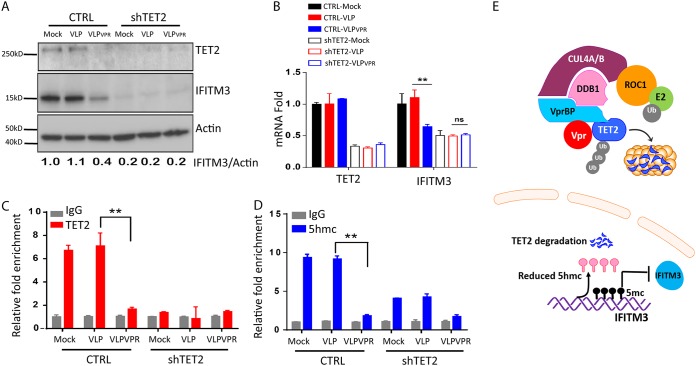
Vpr degrades TET2 to reduce demethylation of the IFITM3 promoter in MDMs. (A and B) TET2 is required for the constitutive expression of IFITM3 in MDMs. (A) Western blot analysis of cell lysates was performed with anti-TET2 or anti-IFITM3 antibody. Relative IFITM3 expression is calculated as IFITM3/actin ratios. (B) MDMs with control or TET2 shRNA were treated with VLPs with or without Vpr for 24 h. TET2 and IFITM3 mRNA levels were detected by RT-qPCR. (C and D) TET2 binds to the IFITM3 promoter to catalyze its demethylation. (C) MDMs transduced with control or TET2 shRNA were treated with VLPs with or without Vpr for 24 h. The ChIP assay was performed with anti-TET2 monoclonal antibody (MAb) to determine TET2 binding to the IFITM3 promoter. (D) ChIP with anti-5hmC MAb was performed to determine demethylation at the IFITM3 promoter. Specific IFITM3 promoter DNA by ChIP is normalized to the DNA ChIP with control IgG. DNA ChIP with control IgG is defined as 1. * and ** indicate *P* < 0.05 and *P* < 0.01, respectively. (E) Schematic diagram of TET2-mediated demethylation of the IFITM3 promoter.

To explore the mechanism of the Vpr-TET2 axis on IFITM3 expression in MDMs, MDMs were treated with vesicular stomatitis virus G protein (VSV-G) pseudotyped virus-like particles (VLPs) in the presence or absence of Vpr. As we reported previously (15), Vpr reduced only TET2 protein but not mRNA (Fig. 5A). However, both mRNA and protein levels of IFITM3 were decreased in MDMs treated with Vpr^+^ VLPs or when TET2 was depleted (Fig. 5A and B), indicating that TET2 increased IFITM3 expression in MDMs. We further performed chromatin immunoprecipitation-quantitative PCR (ChIP-qPCR) experiments to measure the binding of TET2 and its demethylated 5hmC level to the IFITM3 promoter in control MDMs and TET2 knockdown MDMs, after treatment with Vpr^+^ or Vpr^−^ VLPs. TET2 bound efficiently to the IFITM3 promoter in control or Vpr^−^ VLP-treated MDMs (Fig. 5C). Consistent with TET2 binding to the IFITM3 promoter, an elevated 5hmC level was enriched at the IFITM3 promoter in MDMs (Fig. 5D). Vpr^+^ VLP or shTET2 transduction in MDMs abolished TET2 and dramatically reduced 5hmC levels at the IFITM3 promoter (Fig. 5C and D). We conclude that Vpr reduces constitutive expression of IFITM3 in MDMs by inhibiting TET2-mediated demethylation of the IFITM3 promoter (Fig. 5E).

### The Vpr-TET2 axis enhances HIV-1 replication in MDMs by reducing IFITM3 expression and by sustaining IL-6 induction after HIV-1 infection.

**FIG 6:**
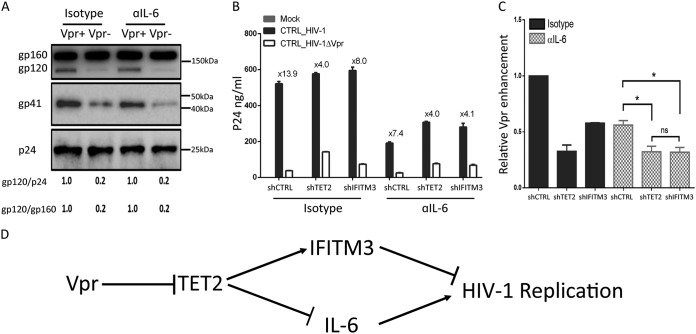
The Vpr-TET2 axis independently enhances HIV-1 replication through reduced IFITM3 and sustained IL-6 expression. (A) Vpr-enhanced Env processing is independent of IL-6 signaling. MDMs were infected with HIV-1 or HIV-1 ΔVpr viruses for 4 days, with NVP added at 2 dpi, and treated with the isotype control or anti-IL-6 neutralizing antibody. Western blots were performed with cell lysates. Env proteins gp160 and gp120 were detected with anti-gp120 antibody, gp41 was detected by anti-gp41 antibody, and p24 was detected by anti-p24 antibody. gp120/p24 and gp120/gp160 ratios are shown. (B) MDMs transduced with control shRNA, shTET2, and shIFITM3 were infected with HIV-1 or HIV-1 ΔVpr and treated with the isotype control or anti-IL-6 neutralizing antibody. p24 levels in the supernatant were measured at 6 dpi. (C) Relative ability of Vpr to enhance HIV-1 replication in cells treated with the isotype control and anti-IL-6 neutralizing antibody at day 6. Data from two different donors are summarized by setting Vpr-enhanced replication in isotype control MDMs transduced with control shRNA at 1.0. * indicates *P* < 0.05. (D) The Vpr-TET2 axis enhances HIV-1 replication in macrophages independently by modulating expression of IFITM3 and IL-6 during HIV-1 infection.

We recently reported that Vpr degrades TET2 to prolong IL-6 expression and enhance viral replication in MDMs (15). To test whether the elevated IL-6 contributes to Vpr-enhanced HIV-1 Env processing in MDMs, we infected MDMs with Vpr^+^ HIV-1 and Vpr^−^ HIV-1 viruses and treated cells with an IL-6 neutralizing antibody for 4 days, with NVP added at 2 days postinfection. As shown in Fig. 6A, IL-6 signaling had no effect on Vpr-enhanced Env processing and no effect on the first round of HIV-1 replication. We further analyzed spreading HIV-1 infection of Vpr^+^ and Vpr^−^ HIV-1 in MDMs for 6 days with depletion of TET2 or IFITM3 treated with IL-6 neutralizing antibody. As we have previously reported (15), depletion of TET2 in MDMs elevated HIV-1 replication and reduced the relative activity of Vpr to enhance HIV replication by 70% (from 13.9× to 4.0×). Interestingly, either IFITIM3 depletion or IL-6 blockade partly reduced the relative Vpr activity to enhance HIV-1 replication in MDMs, and the combination of IFITIM3 depletion with IL-6 blockade was similar to TET2 depletion in the reduction of Vpr-enhanced HIV replication (Fig. 6B and C; see Fig. S4 in the supplemental material). These results suggest that the Vpr-TET2 axis enhances HIV-1 replication in MDMs both by reducing IFITIM3 expression and by sustaining IL-6 induction, and the virions with higher infectivity and elevated IL-6 independently enhance HIV-1 replication after the first round of infection (Fig. 6D).

10.1128/mBio.01344-19.4 FIG S4 The Vpr-TET2 pathway enhances HIV-1 replication through both IFITM3 and IL-6 signaling. HIV-1 or HIV-1 ΔVpr was used for infection of MDMs transduced with control shRNA, shTET2, and shIFITM3 and treated with isotype control and IL-6 neutralizing antibody. p24 levels were measured on the indicated days postinfection. (A) Donor 1. (B) Donor 2. For related details, see Fig. 6. Download FIG S4, PDF file, 0.3 MB. Copyright © 2019 Wang and Su. 2019 Wang and Su This content is distributed under the terms of the Creative Commons Attribution 4.0 International license.

## DISCUSSION

HIV-1 Vpr preferentially enhances HIV-1 replication after the first round of infection in human macrophages via multiple mechanisms that are not clearly defined. We have recently discovered that Vpr targets the DNA demethylase TET2 for degradation, which leads to sustained IL-6 expression and elevated second-round HIV-1 infection. We demonstrated here that Vpr enhanced Env processing, associated with increased virion infectivity during the first round of infection in macrophages. We found IFITIM3 was constitutively expressed in macrophages in a TET2-dependent fashion. Vpr-enhanced Env processing depended genetically on TET2 and IFITM3. We further showed that Vpr suppressed IFITM3 expression by reducing its promoter demethylation in macrophages, associated with degradation of TET2 and reduced TET2 binding to the IFITIM3 promoter. We conclude that the Vpr-TET2 axis enhances HIV-1 replication in macrophages via two independent mechanisms: reduced IFTIM3 expression to enhance Env processing and virion infectivity and sustained IL-6 expression to increase HIV-1 gene expression.

The functional importance of Vpr was first reported in rhesus macaques infected with Vpr-deficient simian immunodeficiency virus (SIV) (5). However, Vpr-deficient HIV-1 viruses show no significant difference in replication in CD4 T lymphocytes (27–29) or in SCID-hu Thy/Liv mice (30), suggesting Vpr targets host restrictions in myeloid cells to enhance HIV-1 replication. In the improved humanized mouse model with both human T and myeloid cells, Vpr has been reported to enhance early HIV-1 infection in proliferating T cells (31).

Vpr has two widely studied activities: one is its ability to induce G_2_ cell cycle arrest, and the other is to increase HIV-1 replication in macrophages (32, 33). The molecular mechanism of G_2_ arrest induction by Vpr has been attributed to a number of host target proteins (34, 35). In one report, Vpr is found to target MUS81 for degradation through the CRL4^VprBP^ E3 ligase, resulting in premature activation of the SLX4 complex. This enhances the cleavage of DNA by SLX4, leading to activation of the DNA damage response and cell cycle arrest (14). We have recently reported that Vpr could enhance HIV-1 replication in MDMs through degradation of TET2 via its activity independent of the G_2_ arrest activity. We have shown that blocking IL-6 signaling partially reduces the ability of Vpr-mediated TET2 degradation to enhance HIV-1 replication, which suggests that the Vpr-TET2 axis has an additional role in enhancing HIV-1 replication in MDMs (15).

In agreement with other studies (6), we found Vpr did not enhance HIV-1 virion production in the first-round infection in MDMs but enhanced HIV-1 Env processing to produce more infectious HIV-1 virions for the second-round infection (Fig. 1). During HIV-1 infection, Env is synthesized as gp160 and is subsequently cleaved in the Golgi apparatus by cellular proteases into gp120 and gp41. The expression, processing, plasma membrane localization, and preferential incorporation of trimeric gp12/gp41 Env into HIV-1 virions have been extensively investigated (36). It has been reported that processed HIV-1 gp120/gp41, but not gp160, is preferentially incorporated into virions via MA-dependent mechanisms (37, 38). Our current results are consistent with this model, showing that impaired gp160 cleavage in the absence of Vpr leads to reduced levels of gp120/gp41 and thus lower levels of gp120/gp41 in the virion (24, 39–42). As we have previously reported, reduced gp120/gp41 in the virions led to reduced infectivity of virions (defined as infectious activity per virion particle) (43, 44).

IFITM proteins, especially IFITM2 and IFITM3, have been reported to bind HIV-1 Env and interfere with its processing (24). We demonstrated that of the 19 reported HIV-1 restriction factors, 6 were expressed efficiently in MDMs, but only IFITM3 was expressed uniquely via TET2-dependent mechanisms (Fig. 2). By degrading TET2, Vpr enhanced Env processing through reduction of IFITM3 in macrophages or MDMs (Fig. 3 and 4). This was confirmed with two macrophage-tropic HIV-1 isolates with CXCR4/CCR5 dualtropic (HIV-R3A [Fig. 1 and 3]) and CCR5-tropic Yu-2 (Fig. 4). Besides inhibiting HIV-1 Env processing, IFITM3 has also been reported to restrict entry of multiple viruses, including HIV-1 (45). Consistently, stable depletion of TET2 and IFITM3 in macrophages also led to increased HIV-1 entry, and Vpr had no further effect on Env processing or infectivity (Fig. 3 and 4; Fig. S3). The TET2-IFITM3 axis in MDMs thus suppresses HIV-1 replication both at the Env processing/virion infectivity step and possibly at the viral entry step in cells previously exposed to Vpr.

IFITM3 expression is regulated by DNA methylation at the IFITM3 promoter (46). We found TET2 bound to the IFITM3 promoter and TET2 catalyzed the conversion of 5-methylcytosine (5mC) to 5-hydroxymethylcytosine (5hmC) on the IFITM3 promoter in MDMs (Fig. 5). Therefore, Vpr-mediated degradation of TET2 in MDMs leads to reduced IFITM3 expression through reduced promoter demethylation. We have recently reported that Vpr-induced degradation of TET2 in macrophages prevents the resolution of IL-6 induction and leads to elevated HIV-1 replication (15). We showed here that IL-6 was not involved in Vpr-enhanced Env processing and infectivity. The Vpr-TET2 axis appears to enhance HIV-1 replication in MDMs independently via reducing IFITM3 expression and sustaining IL-6 expression (Fig. 6). The Vpr-TET2 axis enhances HIV-1 infection in MDMs by reducing IFITM3 expression via a TET2 dioxygenase-dependent mechanism and by sustaining IL-6 induction via TET2 dioxygenase-independent mechanisms. It is likely that Vpr still possesses TET2-indepedent activity that can further enhance HIV-1 infection in macrophages, as suggested by the partial Vpr activity to enhance HIV replication in TET2-depleted cells (Fig. 6C). The Vpr-TET2 axis may provide a novel target to develop anti-HIV drugs to inhibit HIV-1 infection and pathogenesis.

## MATERIALS AND METHODS

### Cell cultures and shRNA.

Human buffy coats were obtained from Gulf Coast Regional Blood Center. Primary human peripheral blood mononuclear cells (PBMCs) were isolated from buffy coats using a Ficoll-Paque gradient. Primary monocytes were isolated from PBMCs using negative magnetic selection (EasySep human monocyte isolation kit 19359). Purified monocytes were seeded at a concentration of 1 × 10^6^ cells/ml in complete RPMI and differentiated into monocyte-derived macrophages (MDMs) using 100 ng/ml granulocyte-macrophage colony-stimulating factor (GM-CSF [Peprotech, Princeton, NJ, USA]) for 6 days. The medium was changed every 2 days throughout 6 days of the culture. TZM-bl was obtained from the NIH AIDS Research and Reference Reagent Program (NIH, Bethesda, MD, USA). TZM-bl was maintained in Dulbecco’s modified Eagle’s medium (DMEM) supplemented with 10% heat-inactivated fetal bovine serum, 100 U/ml penicillin, and 100 μg/ml streptomycin. MAGI/X4R5-cells are maintained in our lab, and the MAGI assay was carried out as previously reported (47). Briefly, MAGI/X4R5 indicator cells were plated in 6-well plates at 1.6 × 10^5^ cells per well in complete DMEM plus G418-hygromycin-puromycin 1 day before HIV-1 infection. Two days after infection, the infected cells were fixed, 600 μl of staining solution was added to each well, and the cells were incubated for 50 min at 37°C. The staining was stopped by removing the staining solution and washing the cells thoroughly twice with phosphate-buffered saline (PBS). Positive blue dots were counted to determine HIV-1 infectious units and viral infectivity.

pGIPZ lentiviral vectors expressing control (nontargeting), TET2-targeting shRNA were purchased from Dharmacon, GE Healthcare (Lafayette, CO). shRNA targeting human TET2 has the mature antisense sequence TAAGTAATACAATGTTCTT (clone ID V3LHS_363201). shRNA targeting human IFITM3 has the mature antisense sequence TGAGCATCTCATAGTTGGG (clone ID V3LHS_325105). Lentiviruses expressing shRNA were produced by CaCl_2_-BES [bis(2-hydroxy)ethylamine] transfection in 293T cells (10-cm plate) using 15 μg pGIPZ-shRNA vector, 10 μg packaging construct ΔNRF, and 5 μg pCMV-VSV-G. The titer of lentiviruses was calculated on 293T cells. Briefly, the day before transduction, a 96-well tissue culture plate was seeded with 293T cells at 2.5 × 10^4^ cells/well in 100 μl of DMEM complete growth medium. Twenty-four hours later, the culture medium was gently removed from each well and a 5-fold serial dilution of viral stock was added to each well with triplicates. This was then spun down for 2 h at 1,500 × *g* at 37°C. The viral stock was removed, and DMEM complete medium was added for 3 days. The lentivirus titer was calculated based on the number of green fluorescent protein (GFP)-expressing cells detected by flow cytometry. For transduction of primary MDMs, 50 ng of VLP-Vpx was used to treat 4 × 10^5^ MDMs for 6 h before lentivirus transduction, and then the cells were washed once with PBS and incubated with GIPZ-shRNA lentivirus (100 ng of p24). All lentiviral transductions were performed by adding 8 μg/ml Polybrene and then spin inoculation of the cells for 2 h at 1,500 × *g* at 37°C. Transduced MDM cells after 3 days were selected with 1 μg/ml puromycin for 3 to 7 days. The transduction efficiency was assessed by GFP expression. All MDMs from different donors presented in the data have achieved >90% transduction efficiency. Knockdown efficiency of TET2 and IFITM3 was subsequently measured by reverse transcription (RT)-qPCR.

### HIV-1 proviral constructs and virion stock production.

X4/R5 dualtropic HIV-1 R3A was generated by our lab as previously described (48). Vpr mutant R3A was generated by insertion of heat-stable antigen (HSA) reporter into the Vpr gene (15). The proviral infectious clone of the macrophage-tropic virus isolate Yu-2 was from the National Institutes of Health AIDS Research and Reference Reagent Program. The same clone disrupted for the Vpr gene, Yu-2 ΔVpr, was generated by PCR to insert two stop codons within the Vpr gene without altering the Vif gene, using the following set of primers: forward, 5′-GATAGATGGAATAAGCCCCAGAAGACTAAGGGCCACAGAGG-3′; and reverse, 5′-CCTCTGTGGCCCTTAGTCTTCTGGGGCTTATTCCATCTATC-3′ (49). HIV-1 virions were produced by CaCl_2_-BES transfection of proviral plasmids in 293T cells. 293T cells cultured on a 10-cm plate were transfected with 30 μg DNA of proviral plasmid for production of replication-competent HIV-1 viruses. For the production of virus-like-particles (VLPs) carrying or not carrying Vpr, we transfected 293T cells with 6 μg p3XFLAG-CMV-Vpr or empty vector, 15 μg pMDLg/pRRE, 4 μg pRSV-Rev, and 5 μg pCMV-VSV-G. For the production of VLP-Vpx particles, we used a Gag/Pol-expressing vector, pMDL-X, containing the Vpx-packaging motifs in the p6 region, as described before (50). VLPs were harvested at 48 h posttransfection and purified by ultracentrifugation (20,000 × *g*, 4°C, 2 h). Concentrations of virion stocks were quantified by p24 ELISA (Frederick National Laboratory for Cancer Research—AIDS and Cancer Virus Program).

### HIV-1 infection.

HIV-1 infection experiments were typically conducted at a multiplicity of infection (MOI) of 0.1, unless otherwise indicated in the figure legends. All HIV-1 infections were typically performed by spin inoculation for 2 h at 1,500 × *g* at 37°C in medium containing 8 μg/ml Polybrene. Virus input was removed after spin inoculation, and fresh medium was added to infected cells. Nevirapine (5 μM) was added to the cells as indicated in the figure legends and maintained during infection. MDMs were treated with 500 ng of p24 per amount of VLPs by spin inoculation for 2 h at 1,500 × *g* at 37°C in medium containing 8 μg/ml Polybrene.

### ELISAs and antibodies.

ELISA kits for human alpha interferon (IFN-α [Mabtech; 3425-1H-20]) were used according to the manufacturer’s instructions. ELISA kits for p24 (Frederick National Laboratory for Cancer Research—AIDS and Cancer Virus Program) were used according to the instructions of the manufacturer. For intracellular p24 detection, infected cells were first permeabilized using Cytofix/Cytoperm from BD following the manufacturer’s protocol and then incubated with anti-p24–fluorescein isothiocyanate (FITC) antibody (1:50 dilution [Beckman Coulter; KC-57]). Anti-TET2 was obtained from Millipore (MABE462). Anti-IFITM3 (11714-1) was obtained from Proteintech. Anti-CD14 was obtained from Abcam (ab181470). Antiactin was obtained from Santa Cruz Biotechnology (sc-47778). Anti-gp120 (catalog no. 288), anti-p24 (catalog no. 6458), anti-gp41 (catalog no. 11557), and sCD4 (catalog no. 4615) were all obtained from the NIH AIDS Research and Reference Reagent Program (NIH, Bethesda, MD, USA). Five hundred nanograms per ml of the isotype control (R&D [MAB004]) or anti-IL-6 neutralizing antibody (R&D [MAB2061R]) was used for the neutralization assay.

### ChIP assay.

For the ChIP assay, cells were collected in an Eppendorf tube and resuspended in 7.5-ml cold PBS. Cellular proteins and DNA were cross-linked by adding 0.5 ml of 16% formaldehyde into the tube (final concentration, 1%) and incubated at 37°C for 15 min. Cells were centrifuged at 1,000 rpm for 5 min, and cell pellets were resuspended in 5 ml PBS buffer containing 0.125 M glycine and 1× protease inhibitor cocktail (PIC). The cells were then incubated at room temperature with shaking for 5 min to quench unreacted formaldehyde. The reaction mixture was centrifuged, and the cell pellets were quickly rinsed with 10 ml of cold PBS and 1× PIC. Cells were collected after centrifugation and transferred into a new Eppendorf tube with 1 ml cold PBS with 1× PIC, followed by centrifugation at 1,000 rpm for 2 min to pellet the cells. Cell pellets were resuspended in 100 μl of SDS lysis buffer (1% SDS, 10 mM EDTA, 50 mM Tris, pH 8.1, 1× PIC) and incubated on ice for 15 min. DNA was sheared by sonication using a Covaris sonicator (13 min at 4°C). Insoluble material was removed by centrifugation at 12,000 × *g* at 4°C for 10 min. The supernatants were transferred to fresh tubes, and 800 μl ChIP dilution buffer containing 1× PIC, 100 μl Dynabeads, and 10 μl antibody was added to each tube. Mixtures were incubated overnight at 4°C with rotation. Beads were precipitated by centrifugation at 2,000 rpm for 2 min at 4°C. The bead-protein-chromatin complexes were washed in 0.5 ml each of the cold buffers in the following order: (i) low-salt wash buffer, (ii) high-salt wash buffer, (iii) LiCl wash buffer, and (iv) TE (Tris-EDTA) buffer (all buffers from Millipore). All washes were performed by incubating complexes for 3 to 5 min with rotation followed by centrifugation at 2,000 rpm for 2 min. One hundred microliters of ChIP dilution buffer and 1 μl proteinase K were then added into each tube, followed by two incubations—the first at 62°C for 2 h and then a second at 95°C for 10 min—both with constant shaking on an Eppendorf ThermoMixer (700 rpm). Samples were cooled down to room temperature and centrifuged at 2,000 rpm for 2 min at 4°C. Supernatants were carefully transferred to a new tube containing 0.5 ml of binding reagent A. The sample-binding reagent A mixtures were transferred to the spin filter in the collection tube, centrifuged for 30 s at 12,000 × *g*, and decanted. Five hundred microliters of wash reagent B was added to the same spin filter in the collection tube and centrifuged for 30 s at 12,000 × *g*. The aqueous portion was carefully discarded, followed by centrifugation for 30 s at 12,000 × *g*. The spin filter was put into a new collection tube, and 50 μl of elution buffer C (Millipore) was added to the center of the spin filter membrane. After centrifugation for 30 s at 12,000 × g, purified DNA was collected for qPCR analysis. The IFITM3 primers used for qPCR analysis of IFITM3 promoter binding TET2 and 5hmc were as follows: forward, 5′-ATTTGTTCCGCCCTCATCTG-3′; and reverse, 5′-GTTTCGGTTTCTCAACAGTTTCCT-3′. Each ChIP DNA fraction’s threshold cycle (*C_T_*) value was normalized to the IgG DNA fraction’s *C_T_* value (Δ*C_T_*) at the same time point. The IgG value is defined as 1.

### Statistical analysis.

Error bars represent the mean ± standard deviation (SD) from triplicate experiments. All statistical analysis was performed with unpaired Student's *t* test, unless otherwise indicated in the figure legends, and results are considered significant when the *P* value is <0.05. *, **, and *** indicate *P* values of <0.05, <0.01, and <0.001, respectively.
