# Supplementary figures and images for: Vpr Enhances HIV-1 Env Processing and Virion Infectivity in Macrophages by Modulating TET2-Dependent IFITM3 Expression
Source: mBio. 2019 Aug 20;10(4):e01344-19. doi: 10.1128/mBio.01344-19 (PMC6703422; doi:10.1128/mBio.01344-19)

Figure S1

A

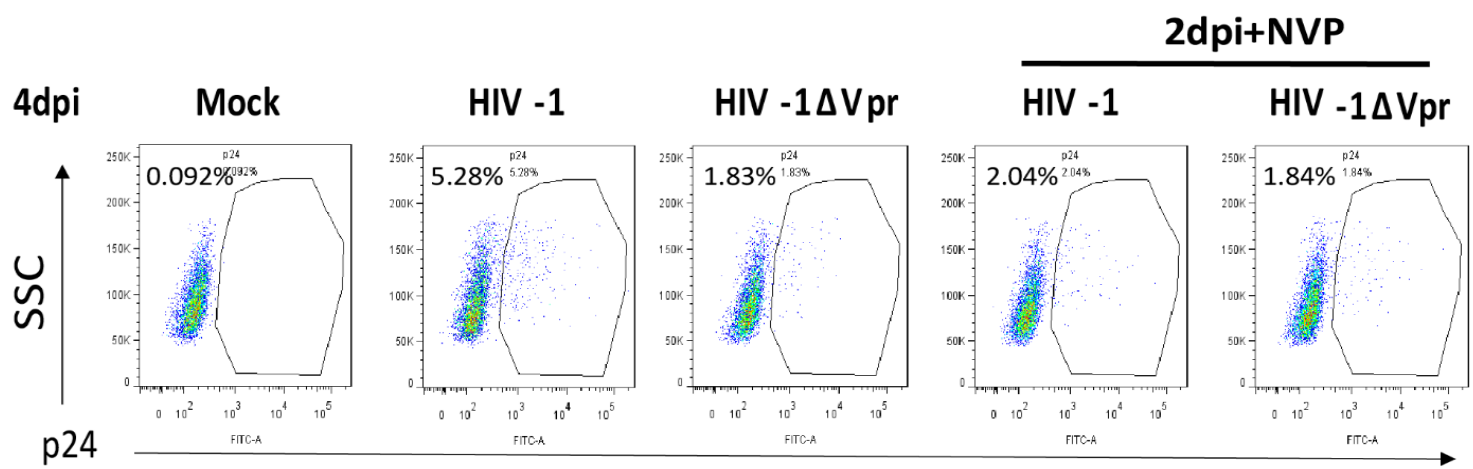

B

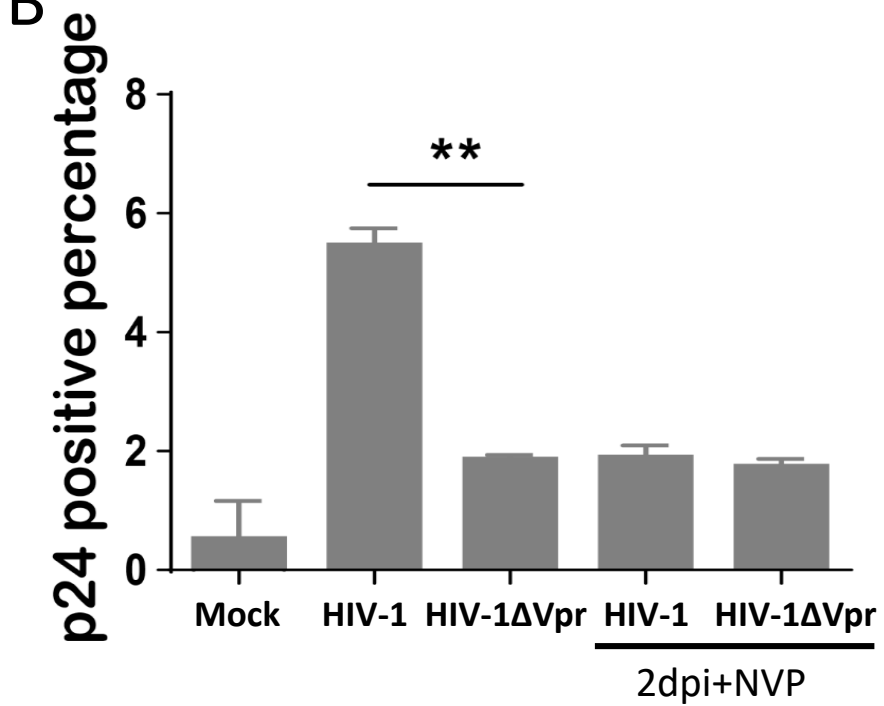

Supplement: FIG S1 [file mBio.01344-19-sf001.pdf]

Figure S2

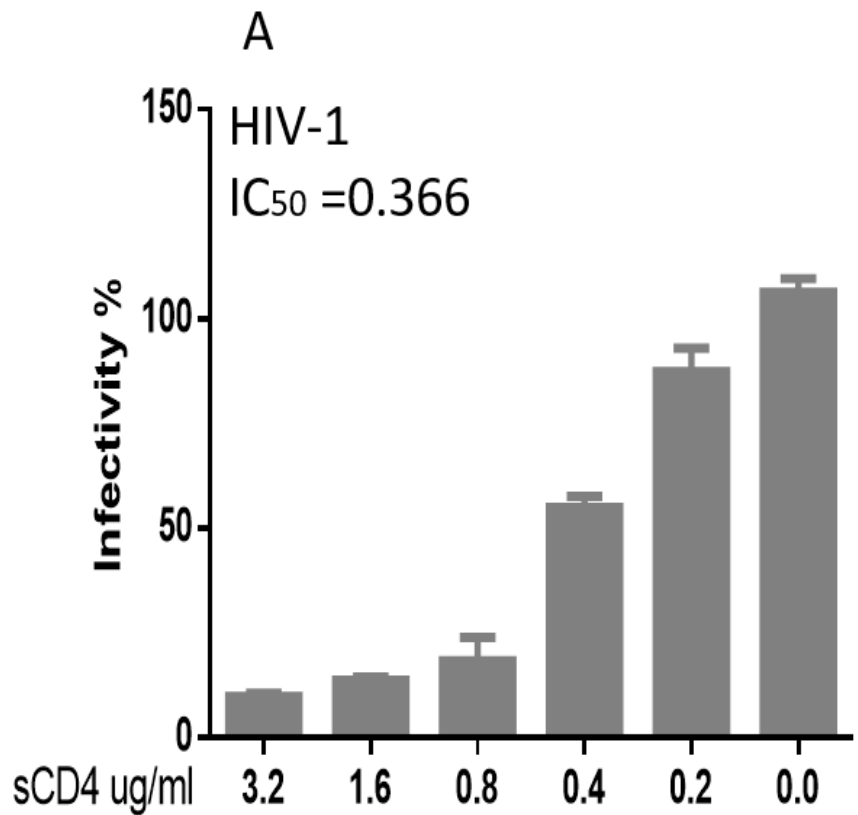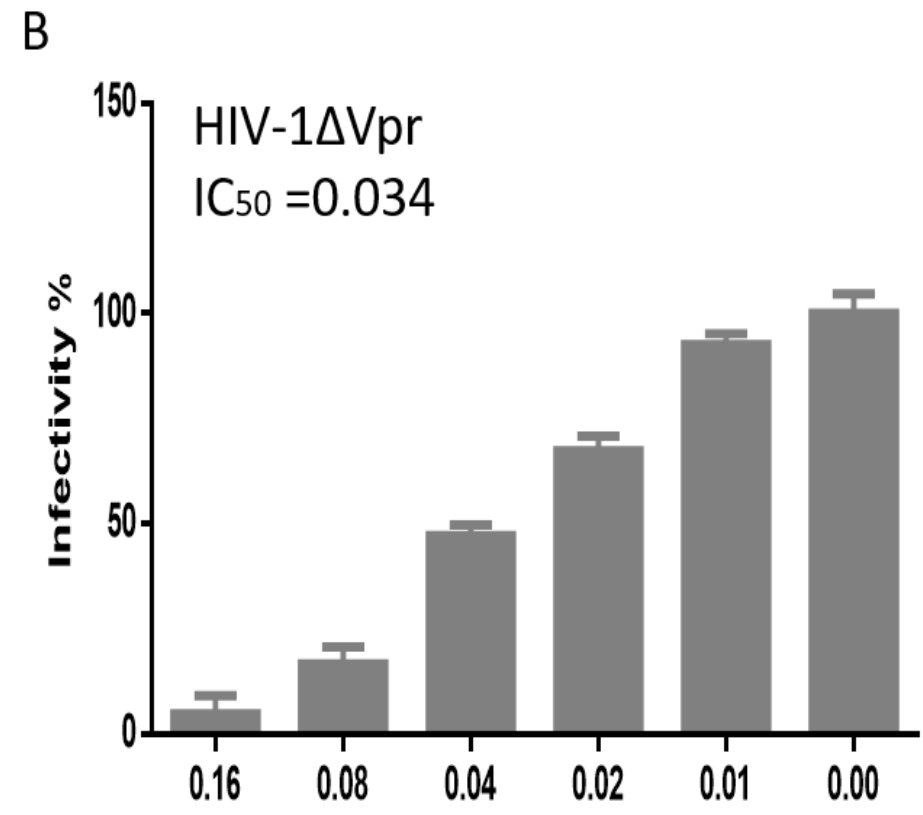

Supplement: FIG S2 [file mBio.01344-19-sf002.pdf]

Figure S3

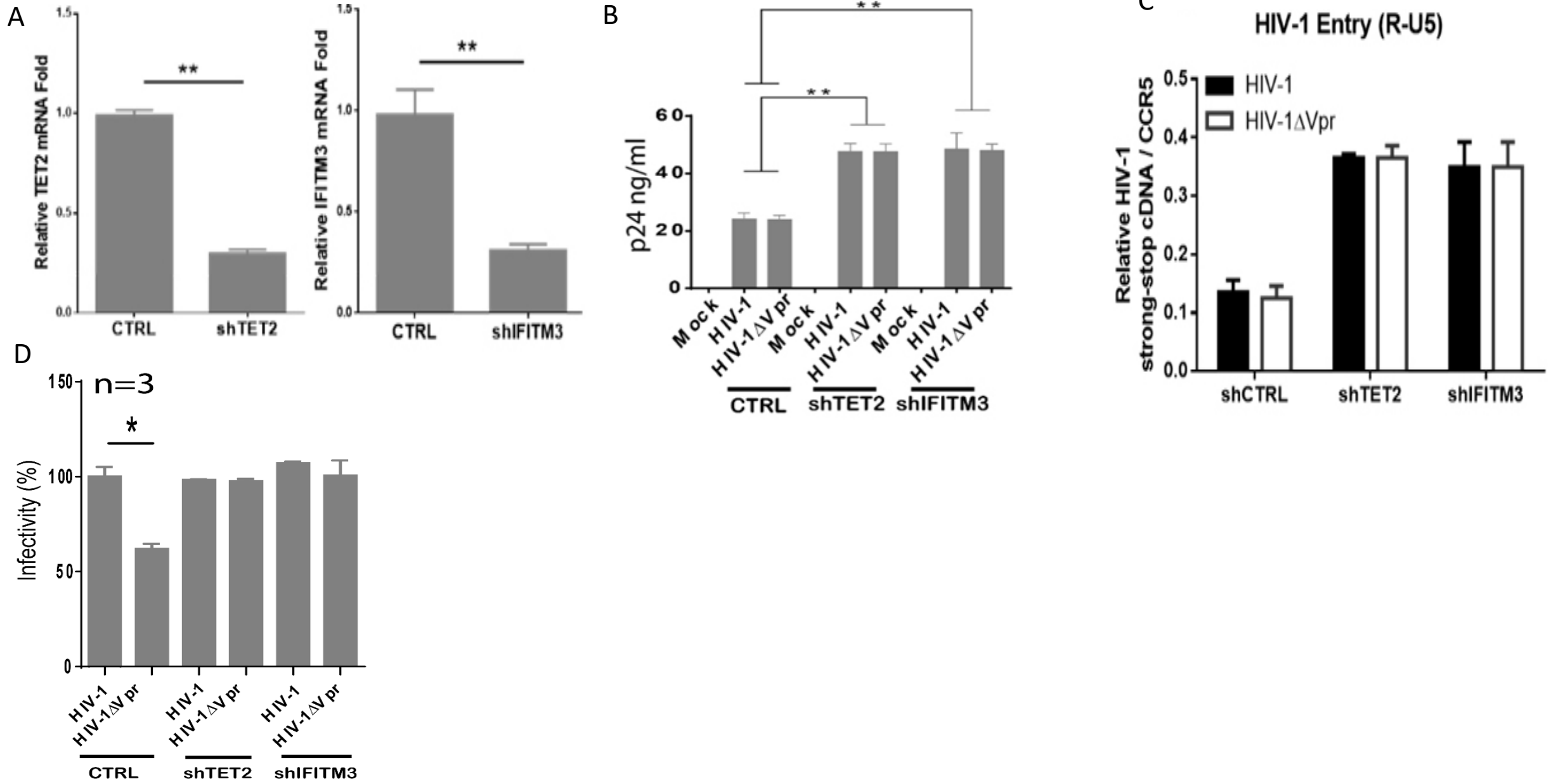

Supplement: FIG S3 [file mBio.01344-19-sf003.pdf]

A

Donor 1

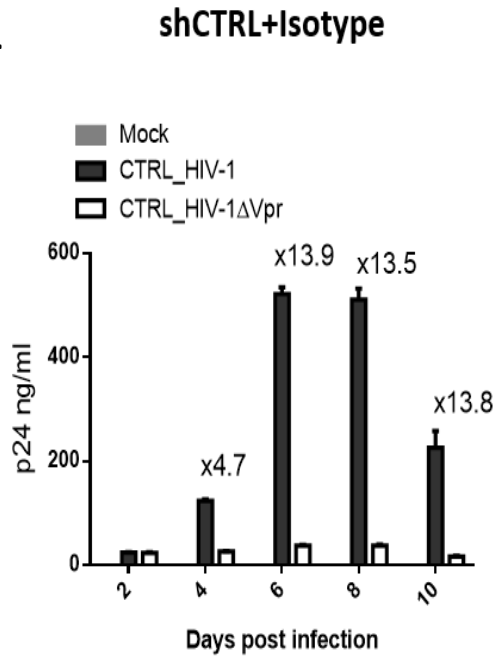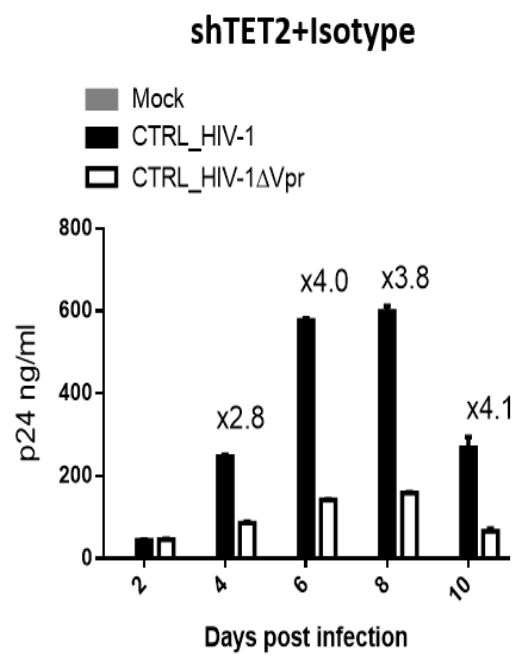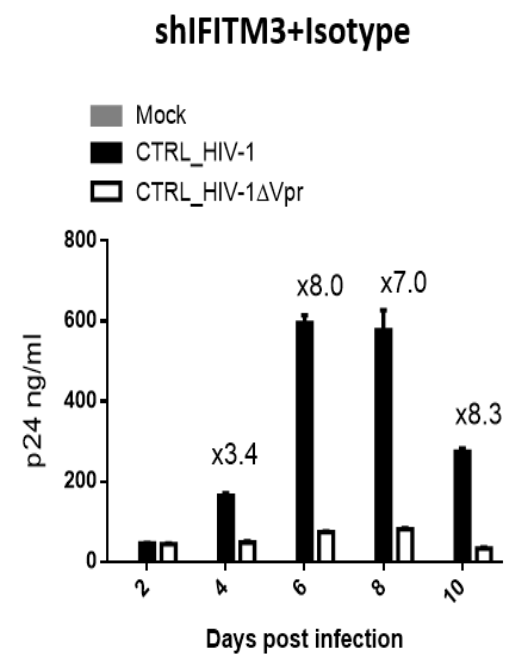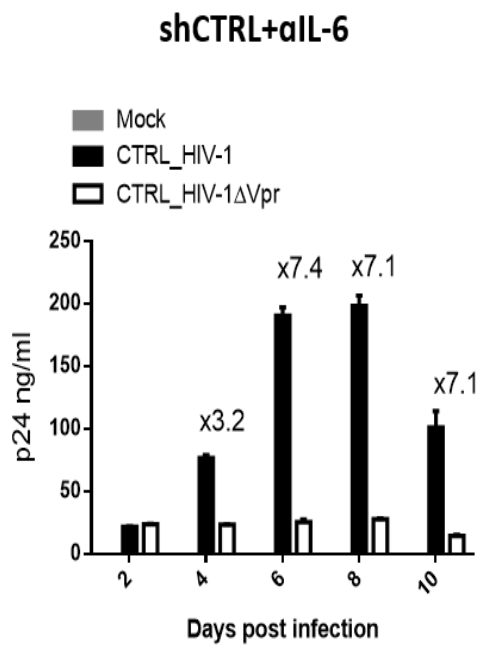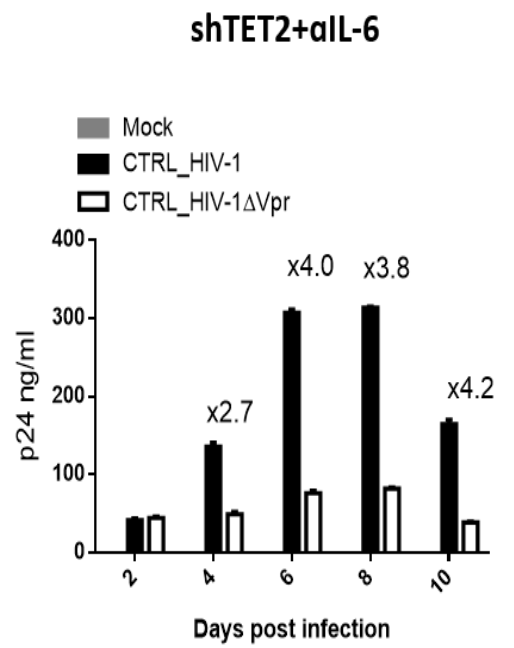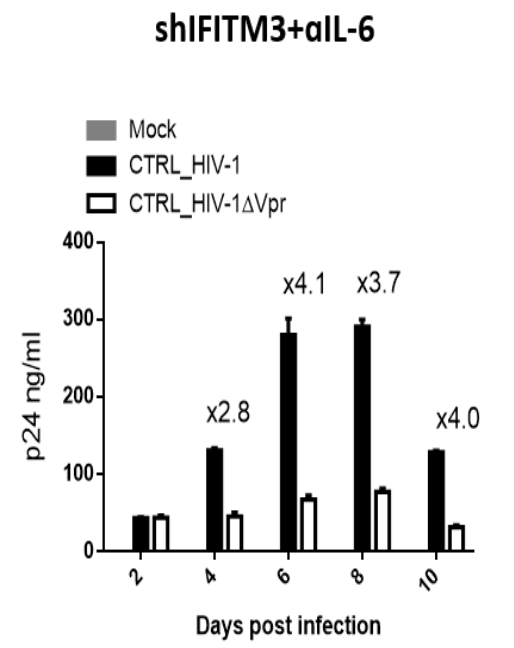

B

Donor 2

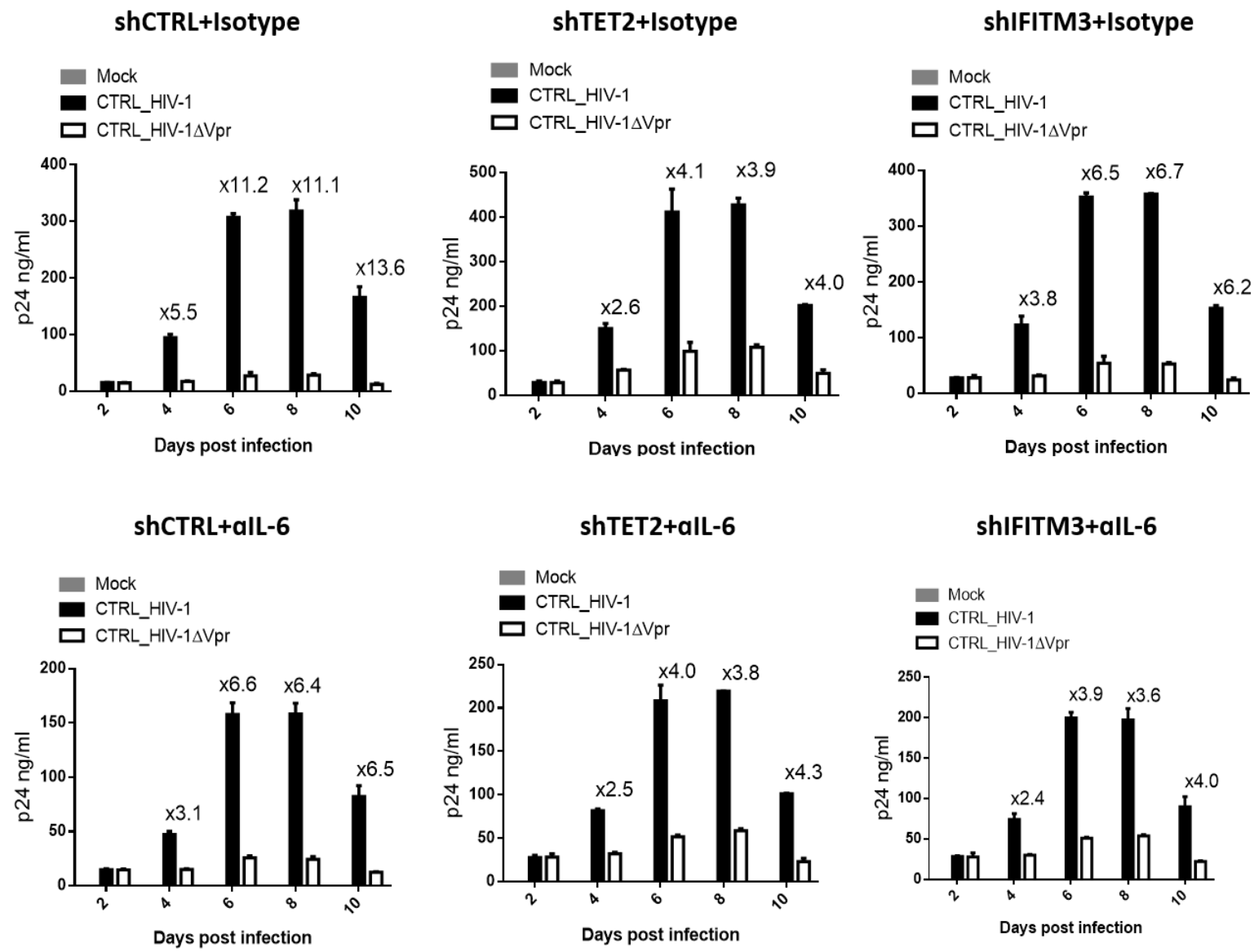

Supplement: FIG S4 [file mBio.01344-19-sf004.pdf]
